# Surgical management of spontaneous, late-onset Descemet membrane detachment after penetrating keratoplasty for keratoconus: a case report

**DOI:** 10.1186/s40662-017-0080-z

**Published:** 2017-06-05

**Authors:** Myrsini Petrelli, Konstantinos Oikonomakis, Konstantinos Andreanos, Andreas Mouchtouris, Ilias Georgalas, George Kymionis

**Affiliations:** Department of Ophthalmology, University of Athens, Athens, Greece

**Keywords:** Keratoconus, Penetrating keratoplasty, Spontaneous, Descemet membrane detachment

## Abstract

**Background:**

To report a surgical method for treating corneal oedema in a case of late-onset Descemet membrane detachment after penetrating keratoplasty.

**Case presentation:**

A 55-year old patient presented with sudden visual loss in his left eye 28 years after penetrating keratoplasty for keratoconus. Slit-lamp biomicroscopy revealed a distortion of the corneal graft anatomy with protrusion of the graft and peripheral thinning and steepening in the residual host tissue, accompanied by corneal graft oedema. Anterior segment optical coherence tomography revealed detachment of Descemet membrane localized to the area of the graft oedema. We proceeded with a full-thickness, partially circumferential incision in the graft-host junction, followed by repositioning and re-suturing of the graft in place, and intracameral air injection in order to achieve reattachment of Descemet membrane.

**Conclusions:**

Corneal graft repositioning in combination with re-bubbling may represent an effective therapeutic option in keratoconic patients with peripheral thinning in the residual host corneal tissue and subsequent Descemet membrane detachment.

## Background

Until recently, repeat-penetrating keratoplasty (PK) has been considered the gold standard for the management of PK graft failure or the recurrence of the primary disease that led to the initial transplantation. Hence, patients had to be re-subjected to various PK-related complications, including delayed wound healing, prolonged visual recovery, high post-operative astigmatism and increased incidence of graft rejection [1, 2]. Currently, Descemet stripping automated endothelial keratoplasty (DSAEK) or Descemet membrane endothelial keratoplasty (DMEK) appear to be a preferred alternative to repeat-PK in cases of PK graft endothelial failure with both satisfactory pre-operative refraction and topography, as well absence of stromal scarring [3]. Unlike PK, DSAEK eliminates suture-related complications and minimizes both post-operative refraction changes and the risk of graft rejection [4]. For these reasons, DSAEK has become the favourable surgical option for the management of endothelial graft failure for numerous corneal surgeons. Nevertheless, a structural stability at the graft-host junction is required in order to achieve regularity of the graft-host interface and subsequently increase graft success and survival.

We present a case of spontaneous Descemet membrane (DM) detachment 28 years after an uneventful PK for keratoconus and report the outcome of a new, modified surgical approach for the treatment of this entity.

## Case presentation

**Fig. 1:**
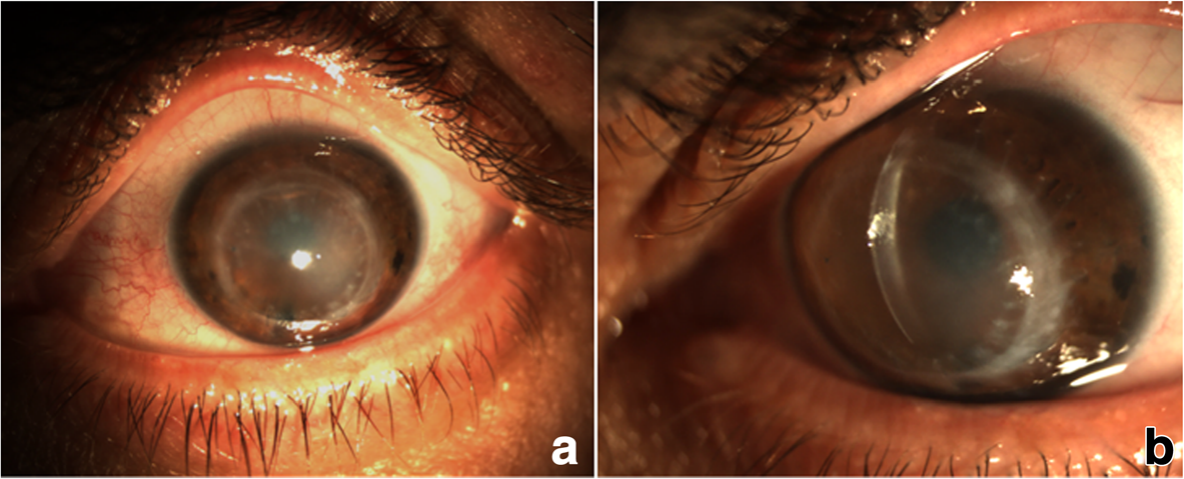
(**a**-**b**). Initial clinical appearance of *left eye*. Slit-lamp photograph showing a bulging, full-thickness graft with diffuse corneal stromal oedema that spares the inferior nasal area

**Fig. 2:**
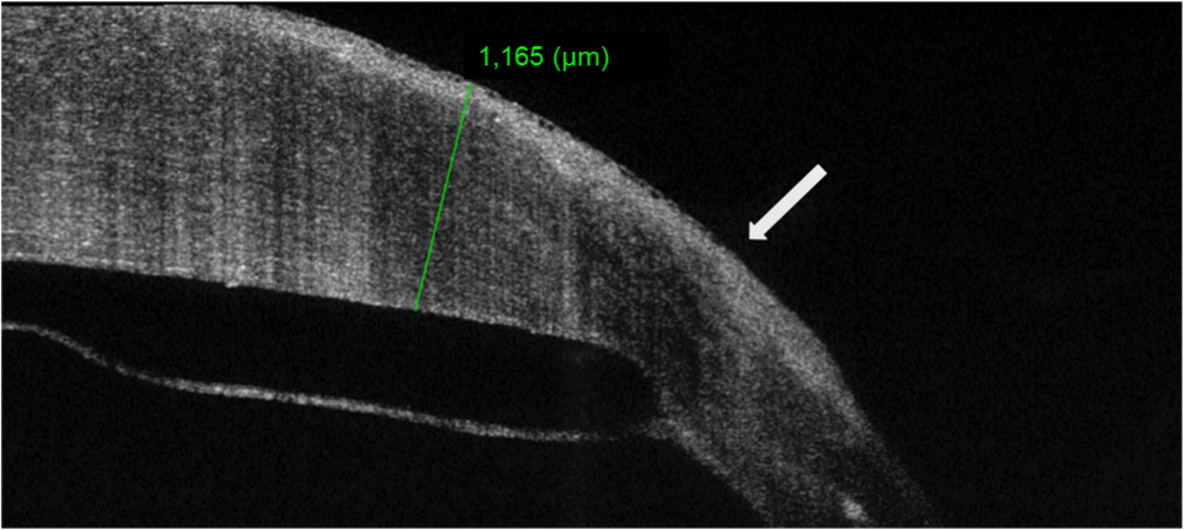
Preoperative anterior segment optical coherence tomography (AS-OCT) of *left eye*. It demonstrates explicitly the Descemet membrane detachment, the overlying corneal graft oedema and graft thickness measurement (1165 μm). *White arrow* points towards the area of corneal thinning

A 55-year old male patient presented to our department complaining of sudden visual loss in his left eye, 28 years after an uncomplicated PK for keratoconus. The patient reported no history of trauma or eye rubbing. Upon presentation, best-corrected visual acuity (BCVA) was 20/40 in the right eye and hand movement (HM) in the left eye. Slit-lamp biomicroscopy showed a bulging, full-thickness graft with distorted curvature and marked peripheral thinning and steepening. Diffuse corneal stromal oedema was observed (9–6 o’clock) that spared the inferior nasal area of the graft (Fig. 1). No signs of infection, graft rejection or failure were identified. Anterior segment optical coherence tomography (AS-OCT, DRI OCT Triton: Topcon Corporation, Tokyo, Japan) revealed a DM detachment, with no DM breaks, localized to the area of the marked oedema (Fig. 2).

We decided to proceed with corneal graft repositioning and re-bubbling. More specifically, a circumferential, full-thickness incision in the previous graft-host junction was made, using fine corneal scissors. The incision was extended for 270^o^ degrees (9 o’clock hours), surrounding the area of the corneal oedema and the underlying detached Descemet membrane. Following this, the graft was repositioned and sutured into place using interrupted 10-0 Nylon sutures. Lastly, air was injected in the anterior chamber in order to achieve reattachment and promote adherence of the previously detached DM.

**Fig. 3:**
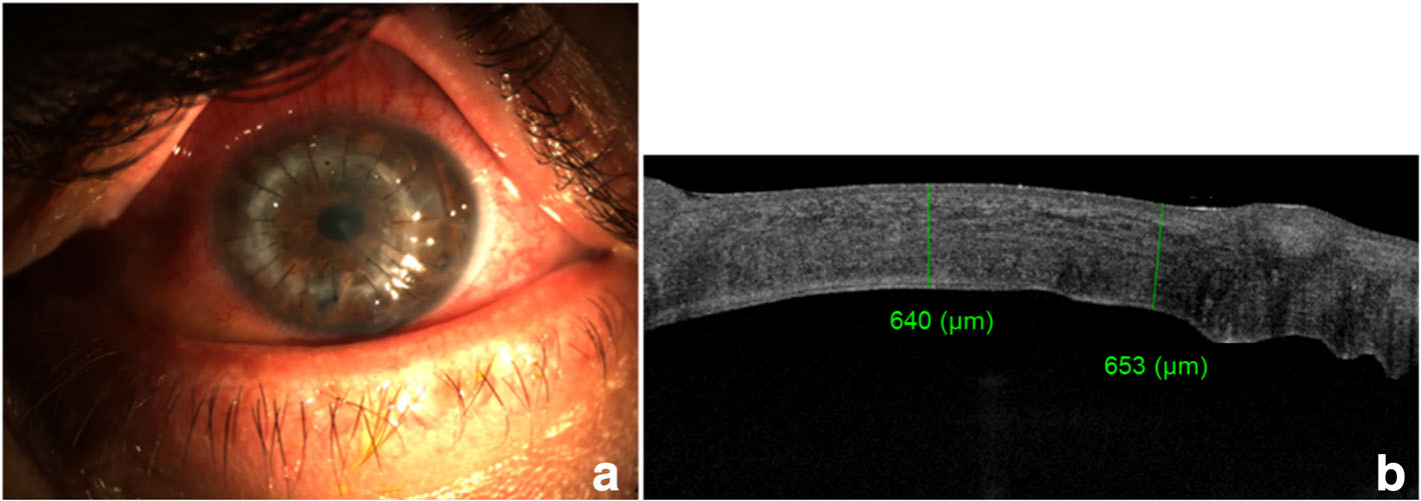
Three-month postoperative appearance of *left eye*. **a** Slit-lamp photograph and **b** anterior segment optical coherence tomography (AS-OCT) showing resolution of the corneal graft oedema (graft thickness is measured to be 640 and 653 μm) and restoration of the graft-host junction structural anatomy

On the first post-operative day, corneal oedema had resolved and DM was found reattached. Patient’s BCVA was 20/40 in the left eye. The postoperative course was uneventful and the graft remained clear after a follow-up of 3 months (Fig. 3a). AS-OCT showed successful graft repositioning and a fully attached DM (Fig. 3b).

## Discussion and Conclusions

Spontaneous DM detachment represents a recently described clinical entity. To our knowledge, this is the third case of spontaneous, late-onset DM detachment after uncomplicated PK to be reported in literature [5]. Previous reports involved two cases of spontaneous DM detachment, 21 and 25 years following penetrating keratoplasty for keratoconus. The authors proposed two possible mechanisms that could explain this phenomenon. The first one included the presence of a retrocorneal membrane that could have caused a mechanical detachment of the DM. The second mechanism involved possible recurrence of keratoconus in the peripheral corneal host tissue. Both patients were ultimately treated with DSAEK.

In our case, similar to previous reports, the corneal graft showed neither scarring nor signs of rejection. The existing endothelial dysfunction and subsequent oedema were attributed to the spontaneous DM detachment. For this reason, re-bubbling or posterior lamellar keratoplasty, such as DSAEK, might have appeared as a more favourable surgical option. Nevertheless, significant thinning and steepening in the peripheral corneal host tissue was observed in our case. This observation was suggestive of possible progression of keratoconus in the residual host tissue. This finding might had complicated any attempt of performing DSAEK, as well as increased the chance of DSAEK failure. Therefore, we decided to attempt restoration of the structural anatomy of the corneal surface at first, by repositioning the graft, and then proceed with re-bubbling. This combination of revisional surgery and re-bubbling may not only have improved the observed peripheral corneal thinning and prevented its further progression, but also increased the possibility of successful DM reattachment. Furthermore, since peripheral thinning and steepening seem to have been managed successfully, as shown by postoperative AS-OCT, and patient’s visual acuity has improved significantly, further improvement in his refractive profile is expected, especially following future removal of sutures. Nevertheless, safe and definite results regarding our patient’s postoperative corneal astigmatism may be reported only after suture removal.

Although the exact aetiology for the late changes in keratometric corneal power after PK in patients with keratoconus has not been elucidated, it may include progression of keratoconus in the host rim or recurrence of ectasia in the donor graft [6–8]. Since longer graft survival periods are currently being achieved and long-time follow-ups take place, an increasing number of similar cases might appear in the future [9]. Nevertheless, novel imaging techniques such as AS-OCT contribute to both early identification of such entities like spontaneous DM detachment, and prompt intervention and treatment.

In conclusion, corneal graft repositioning in keratoconic patients with signs of progressive peripheral corneal thinning and subsequent DM detachment, may represent an alternative, effective therapeutic option.

## Abbreviations

AS-OCT: Anterior segment optical coherence tomography
BCVA: Best-corrected visual acuity
DM: Descemet membrane
DSAEK: Descemet stripping automated endothelial keratoplasty
PK: Penetrating keratoplasty
